# Supplementary material for: Manufacture and characterization of graphene membranes with suspended silicon proof masses for MEMS and NEMS applications
Source: Microsyst Nanoeng. 2020 Apr 20;6:17. doi: 10.1038/s41378-019-0128-4 (PMC8433294; doi:10.1038/s41378-019-0128-4)
Supplement: Supplementary file 1 — Supporting Information [file 41378_2019_128_MOESM1_ESM.docx]

**Supporting Information**

**Manufacture and Characterization of Graphene Membranes with Suspended Silicon Proof Masses** **for MEMS and NEMS Applications**

Xuge Fan^1*^, Anderson D. Smith^2^, Fredrik Forsberg^1^, Stefan Wagner^3^, Stephan Schröder^1^, Sayedeh Shirin Afyouni Akbari^4^, Andreas C. Fischer^1,5^, Luis Guillermo Villanueva^4^, Mikael Östling^2^, Max C. Lemme ^3,6*^ and Frank Niklaus^1*^

^1^Division of Micro and Nanosystems, School of Electrical Engineering and Computer Science, KTH Royal Institute of Technology, SE-10044 Stockholm, Sweden.

^2^Faculty of Electrical Engineering and Information Technology, RWTH Aachen University, Otto-Blumenthal-Str. 25, 52074 Aachen, Germany.

^3^Division of Integrated Devices and Circuits, School of Electrical Engineering and Computer Science, KTH Royal Institute of Technology, SE-164 40 Kista, Sweden.

^4^Advanced NEMS Group, École Polytechnique Fédérale de Lausanne (EPFL), 1015 Lausanne, Switzerland.

^5^Silex Microsystems AB, 175 26 Järfälla, Sweden.

^6^AMO GmbH, Advanced Microelectronic Center Aachen (AMICA), Otto-Blumnethal-Str. 25, 52074 Aachen, Germany

Corresponding authors

Name: Xuge Fan, *E-mail: (X.F.) [xuge@eecs.kth.se](mailto:xuge@eecs.kth.se)

Name: Frank Niklaus, *E-mail: (F.N.) frank.niklaus@eecs.kth.se

Name: Max C. Lemme, *E-mail: (M.C.L.) lemme@amo.de


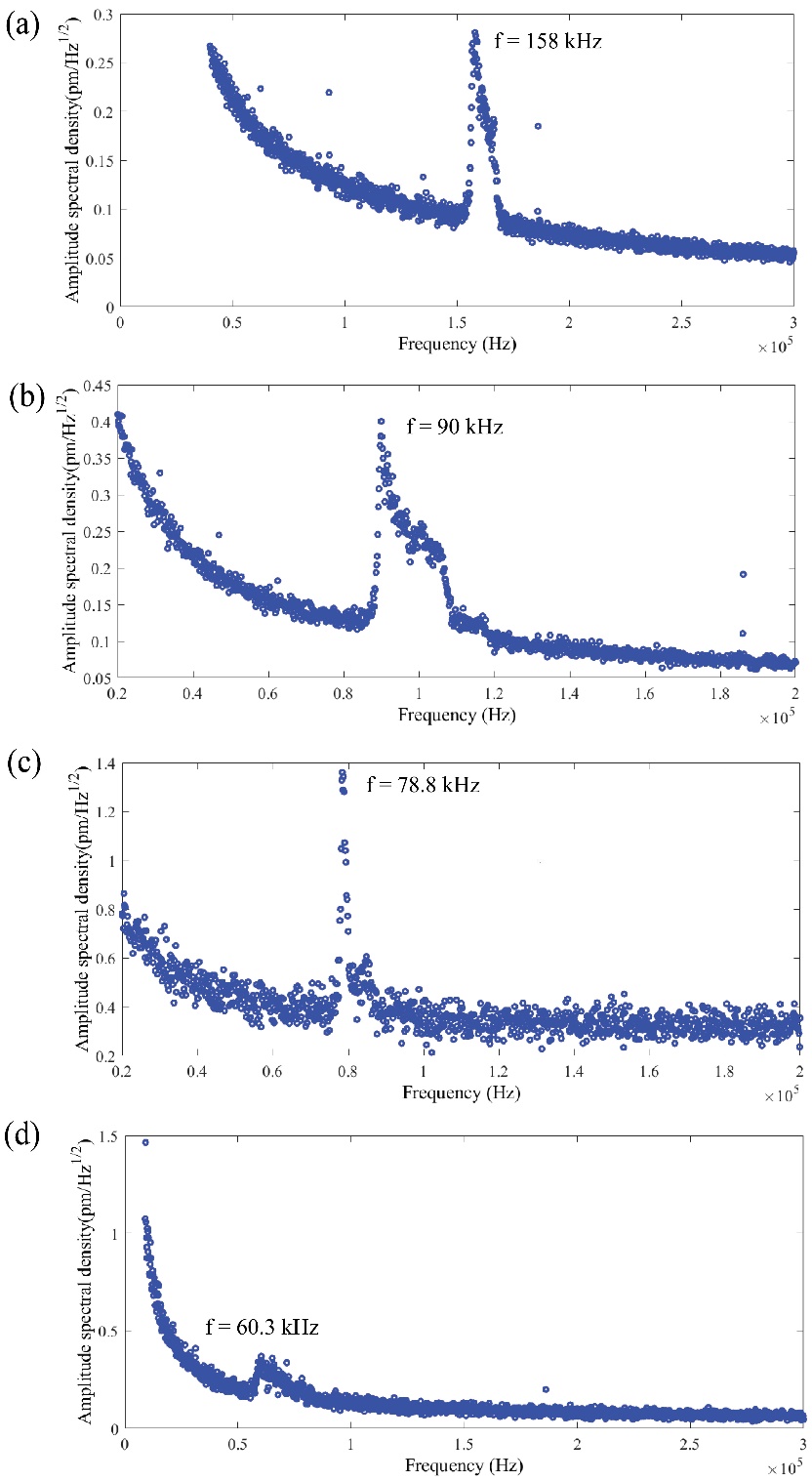


**Figure S1** LDV frequency scans of the four devices shown in Figure 5, with resonance frequencies of (**a**) 158 kHz, (**b**) 90 kHz, (**c**) 78.8 kHz and (**d**) 60.3 kHz, respectively. The four devices have identical trench widths of 3 µm, but different proof mass dimensions of (**a**) 25 µm × 25 µm × 16.4 µm; (**b**) 30 µm × 30 µm × 16.4 µm; (**c**) 40 µm × 40 µm × 16.4 µm and (**d**) 50 µm × 50 µm × 16.4 µm.


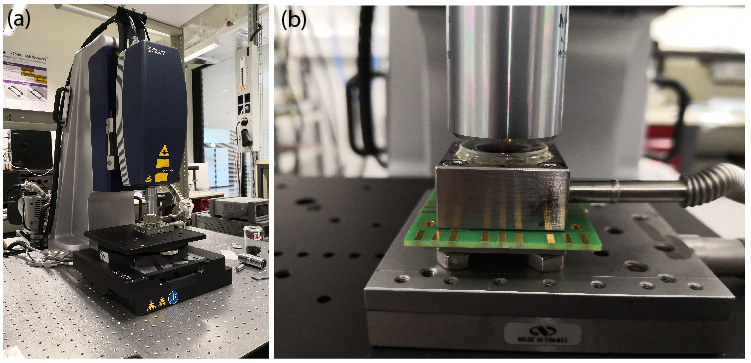
**Figure S2** (**a**) Photo of LDV measurement setup, (**b**) Zoom in of (**a**).

**Table S1 Displacement of the suspended silicon proof mass of a device at different applied AFM indentation forces and estimate of the resulting strain in the graphene membranes.**

| Proof mass size: 20 µm × 20 µm × 16.4 µm; Trench width: 4 µm | | | |
| --- | --- | --- | --- |
| AFM tip indentation force (nN) | Displacement of suspended proof mass (nm) | Status of graphene membrane with suspended proof mass | Estimated equivalent average strain assuming a doubly-clamped graphene ribbon (ε = 2Z^2^/L^2^) * |
| 16 | 8 | Intact | ~1.9 × 10^-6^ |
| 63 | 23 | Intact | ~1.7 × 10^-5^ |
| 159 | 39 | Intact | ~4.8 × 10^-5^ |
| 377 | 94 | Intact | ~3 × 10^-4^ |
| 572 | 127 | Intact | ~5 × 10^-4^ |
| 762 | 154 | Intact | ~7 × 10^-4^ |
| 942 | 163 | Intact | ~8 × 10^-4^ |
| 1145 | 178 | Intact | ~1.0 × 10^-3^ |
| 1318 | 191 | Intact | ~1.1 × 10^-3^ |
| 1511 | 205 | Intact | ~1.3 × 10^-3^ |
| 1880 | 222 | Intact | ~1.5 × 10^-3^ |
| 2648 | 257 | Intact | ~2.1 × 10^-3^ |
| 2835 | 265 | Intact | ~2.2 × 10^-3^ |
| 3200 | 281 | Intact | ~2.5 × 10^-3^ |
| 3585 | 301 | Intact | ~2.8 × 10^-3^ |
| 3777 | 312 | Intact | ~3 × 10^-3^ |
| 4146.5 | 339 | Intact | ~3.6 × 10^-3^ |
| 4523 | 371 | Intact | ~4.3 × 10^-3^ |
| 4712 | 397 | Intact | ~4.9 × 10^-3^ |
| 5091 | 425 | Intact | ~5.7 × 10^-3^ |
| 5459 | 465 | Intact | ~6.8 × 10^-3^ |
| 5650 | 486 | Intact | ~7.4 × 10^-3^ |
| 5964 | 524 | Intact | ~8.6 × 10^-3^ |
| 6481 | 590 | Intact | ~1.1 × 10^-2^ |
| 6968 | 677 | Intact | ~1.4 × 10^-2^ |

* The average strain of a doubly-clamped graphene ribbon can be estimated to ε = 2Z^2^/L^2^, where Z is the deflection at the centre of the suspended ribbon and L is the length of doubly-clamped suspended graphene ribbon. Strain values of a doubly-clamped ribbon with a length of 8 µm can be calculated by using the displacement values of the suspended proof mass caused by AFM tip indentation forces. If the defects (holes) in the suspended graphene membranes are ignored, the average strain values of a graphene membrane in our structures should be approximately equal or smaller than the corresponding average strain values of a graphene ribbon with a length of two times the trench width.

**Table S2: Strain in fully-clamped circular or square graphene membranes by AFM indentation experiments reported in previous literatures.**

| Author | Type | Thickness (nm) | Force  (nN) | Deflection  (Z)  (nm) | Radius  (a)  (µm) | Strain  (excl. built-in strain)  (ε) * |
| --- | --- | --- | --- | --- | --- | --- |
| Lee^1^ | Fully-clamped circular membrane | Single-layer graphene | ~1200 | ~120 | ~0.75 | ~1.7 × 10^-2^ |
| Guillermo^2^ | Fully-clamped circular membrane | Single-layer graphene | ~550 | ~80 | ~0.5 | ~1.7 × 10^-2^ |
| Lee^3^ | Fully-clamped circular membrane | Single-layer graphene | ~2000 | ~95 | ~0.75 | ~1.07 × 10^-2^ |
| Annamalai^4^ | Fully-clamped circular membrane | Single-layer graphene | ~560 | ~128 | ~1.88 | ~3.1 × 10^-3^ |
| Poot^5^ | Fully-clamped circular membrane | Graphene flakes  (~69 layers) | unknown | ~15 | ~0.5 | ~6 × 10^-4^ |
| Bunch^6^ | Fully-clamped square membrane  ** | Single-layer  graphene | ~10.7 | ~30 | ~2.375 | ~1.06 × 10^-4^ |

* The strain for a fully-clamped circular membrane can be estimated to ε = 2Z^2^/3a^2^.

** Fully clamped square membranes were approximated by fully clamped inscribed circular membranes.

**Reference**

1. Lee, C., Wei, X., Kysar, J. W. & Hone, J. Measurement of the elastic properties and intrinsic strength of monolayer graphene. *Science* **321,** 385–388 (2008).

2. López-Polín, G. *et al.* Increasing the elastic modulus of graphene by controlled defect creation. *Nat. Phys.* **11,** 26–31 (2014).

3. Lee, G.-H. *et al.* High-strength chemical-vapor–deposited graphene and grain boundaries. *Science* **340,** 1073–1076 (2013).

4. Annamalai, M., Mathew, S., Jamali, M., Zhan, D. & Palaniapan, M. Elastic and nonlinear response of nanomechanical graphene devices. *J. Micromech. Microeng.* **22,** 105024 (2012).

5. Poot, M. & van der Zant, H. S. J. Nanomechanical properties of few-layer graphene membranes. *Appl. Phys. Lett.* **92,** 063111 (2008).

6. Bunch, J. S. *et al.* Impermeable Atomic Membranes from Graphene Sheets. *Nano Lett.* **8,** 2458–2462 (2008).
